# Supplementary material for: Breastfeeding, Complementary Feeding, Physical Activity, Screen Use, and Hours of Sleep in Children under 2 Years during Lockdown by the COVID-19 Pandemic in Chile
Source: Children (Basel). 2022 Jun 1;9(6):819. doi: 10.3390/children9060819 (PMC9221584; doi:10.3390/children9060819)
Supplement: Supplementary file 1 [file children-09-00819-s001.zip › children-1725999-supplementary.pdf]

Supplementary Table S1. Questions associated with food consumption, physical activity, screen time and hours of sleep in Children under 2 years during Lockdown by the COVID-19 Pandemic in Chile.

Questions	Answer options
**0 to 5 months**	
Up what age did your child ingest or ingest exclusive breastfeeding?	Never received<br>Still receive<br>Until the 1st month of life<br>Until the 2nd month of life<br>Until the 3rd month of life<br>Until the 4th month of life<br>Until the 5th month of life
About to breast milk, has your child eaten or drank any other type of food? (Select the food or foods that she or he has eaten or eats)	You have not eaten or taken any other food<br>Water<br>Herbal waters<br>Fruit juice<br>Fruit puree<br>Yogurt<br>Bread<br>Cookies<br>Candies<br>Other foods
**6 to 11 months**	
Up what age did your child ingest or ingest exclusive breastfeeding?	Never received<br>Still receive<br>Until the 1st month of life<br>Until the 2nd month of life<br>Until the 3rd month of life<br>Until the 4th month of life<br>Until the 5th month of life<br>Until the 6th month of life
At what age did your child start complementary feeding?	It still hasn't started<br>Before 4 months of life<br>At 4 months of life<br>At 5 months of life<br>At 6 months of life<br>After 6 months of life
At what age did your child ingest their second meal (dinner)?	Still not received<br>Before 6 months of life<br>At 6 months of life<br>At 7 months of life<br>At 8 months of life<br>At 9 months of life<br>At 10 months of life<br>At 11 months of life<br>At 12 months of life

At what age did your child eat legumes for the first time (beans, lentils, chickpeas, among others)?	Does not eat<br>At 6 months<br>Between 7 and 8 months<br>Between 9 and 12 months
How many times does your child eat preparations with legumes?	Does not eat<br>1 time per week<br>2 times per week<br>3 or more times a week<br>1 or 2 times a month
At what age did your child first eat fish?	Does not eat<br>Between 6 and 7 months<br>Between 8 and 9 months<br>Between 10 and 12 months
How many times does your child eat preparations with fish?	Does not eat<br>1 time per week<br>2 times per week<br>3 or more times a week<br>1 or 2 times a month
At what age did your child eat an egg for the first time?	Does not eat<br>Between 6 and 8 months<br>Between 9 and 10 months<br>Between 11 and 12 months
Does your child eat sweet snacks (cookies, cakes, chocolates, or candies) or salty ones (chips, Doritos, twigs, among others)?	Every day<br>3 or more times a week<br>2 times a week<br>1 time per week<br>1 or 2 times a month<br>Does not eat
Does your child eat foods to which you add sweeteners such as: stevia, sucralose, saccharin, aspartame, allulose, tagatose or agave?	Every day<br>3 or more times a week<br>2 times a week<br>1 time per week<br>1 or 2 times a month<br>Does not eat
Do you add salt and/or sugar to your child's meals, desserts, or milk?	Only salt<br>Only sugar<br>Salt and sugar<br>Does not add
**12 to 23 months**	
Up what age did your child ingest or ingest exclusive breastfeeding?	Never received<br>Still receive<br>Until the 1st month of life<br>Until the 2nd month of life<br>Until the 3rd month of life<br>Until the 4th month of life<br>Until the 5th month of life<br>Until the 6th month of life

At what age did your child start complementary feeding?	It still hasn't started<br>Before 4 months of life<br>At 4 months of life<br>At 5 months of life<br>At 6 months of life<br>After 6 months of life
At what age did your child ingest their second meal (dinner)?	Still not received<br>Before 6 months of life<br>At 6 months of life<br>At 7 months of life<br>At 8 months of life<br>At 9 months of life<br>At 10 months of life<br>At 11 months of life<br>At 12 months of life
At what age did your child eat legumes for the first time (beans, lentils, chickpeas, among others)?	Does not eat<br>At 6 months<br>Between 7 and 8 months<br>Between 9 and 12 months
How many times does your child eat preparations with legumes?	Does not eat<br>1 time per week<br>2 times per week<br>3 or more times a week<br>1 or 2 times a month
At what age did your child first eat fish?	Does not eat<br>Between 6 and 7 months<br>Between 8 and 9 months<br>Between 10 and 12 months
How many times does your child eat preparations with fish?	Does not eat<br>1 time per week<br>2 times per week<br>3 or more times a week<br>1 or 2 times a month
At what age did your child eat an egg for the first time?	Does not eat<br>Between 6 and 8 months<br>Between 9 and 10 months<br>Between 11 and 12 months
How many times does your child eat raw salads?	Does not eat<br>Daily<br>1 to 3 times a week<br>4 to 6 times a week
Does your child eat sweet snacks (cookies, cakes, chocolates, or candies) or salty ones (chips, Doritos, twigs, among others)?	Every day<br>3 or more times a week<br>2 times a week<br>1 time per week<br>1 or 2 times a month<br>Does not eat

Does your child eat foods to which you add sweeteners such as: stevia, sucralose, saccharin, aspartame, allulose, tagatose or agave?	Every day<br>3 or more times a week<br>2 times a week<br>1 time per week<br>1 or 2 times a month<br>Does not eat
Do you add salt and/or sugar to your child's meals, desserts, or milk?	Only salt<br>Only sugar<br>Salt and sugar<br>Does not add
**All children**	
Does your child watch any kind of screen while eating? (Screen refers to: TV, cell phone, desktop computer, notebook, or tablet)	Always<br>Sometimes<br>Never
How many hours of screens per day does your child watch for entertainment? (Screen refers to: TV, cell phone, desktop computer, notebook, or tablet)	Don't use screens<br>less than 1 hour<br>Between 1 and 2 hours<br>Between 3 and 4 hours<br>More than 4 hours
Does your child engage in some form of physical activity?<br><br>For children under 1 year old: interactive games with parents or caregivers, crawling or turning in their bed.<br>For children between 1 and 2 years old: interactive games with parents or caregivers, crawling, walking alone or with support.	Yes<br>Not
How many hours of physical activity does your child do?	Not done<br>At least 30 minutes daily<br>At least 1 hour daily<br>At least 2 hours a day<br>At least 3 hours a day
How many hours does your child sleep each day (including naps)?	Less than 8 hours<br>Between 9 and 10 hours<br>Between 11 and 14 hours<br>Between 15 and 17 hours<br>More than 17 hours
